# Osteopontin protects from ovalbumin-induced asthma by preserving the microbiome and the intestinal barrier function

**DOI:** 10.1128/msystems.00389-25

**Published:** 2025-05-22

**Authors:** Jinli Huang, Hongyu Qiao, Qiuhong Li, Yi Zhang, Chenyu Zhang, Hui Su, Xin Sun

**Affiliations:** 1 Department of Pediatrics, Xijing Hospital, the Fourth Military Medical University, Xi’an, China; 2 Department of Geriatrics, Xijing Hospital, the Fourth Military Medical University, Xi’an, China; The University of Hong Kong, Hong Kong, Hong Kong

**Keywords:** osteopontin (OPN), asthma, gut and lung microbiota, PD-1/PD-L1 pathway, fecal microbiota transplantation, inflammation, immunity

## Abstract

The gut and lung microbiota are associated with asthma. Osteopontin (OPN) is an important cytokine associated with several inflammatory diseases. The potential role of OPN in the asthma-associated microbiome remains poorly understood. Here, we investigated whether OPN could moderate asthma by affecting the gut and lung microbiota. Our results showed that compared with wild-type (WT) mice, Spp1^−/−^ mice exhibited immune cell infiltration in the lung, OVA-specific IgG1, increased levels of Th2- and Th17-related inflammatory factors, and decreased levels of Th1-related inflammatory factors and forkhead box P3 (FOXP3) expression, resulting in a Th1/Th2 and Th17/Treg imbalance. In addition, gut structure was impaired, and expression of tight junction-related proteins was reduced in Spp1^−/−^ mice, which disrupted gut barrier function. Importantly, OPN-deficient significantly aggravated gut and lung microbiota dysbiosis in OVA-induced asthmatic mice. The results of high-throughput 16S rRNA sequencing demonstrated that OPN-deficient mice showed a substantial reduction in beneficial gut and lung bacteria (*Bacteroidetes*, *Lactobacillus*, *Allobaculum*), and an OVA-induced increase in the abundance of bacteria associated with potentially pathogenic gut and lung (*Epsilonbacteraeota*, *Helicobacter*, *Desulfovibrio*, *Oscillibacter*)-associated bacteria was elevated in abundance. *Allobaculum* was negatively correlated with interleukin-4 and GATA-3 and was positively correlated with interferon gamma and FOXP3. Moreover, through fecal microbiota transplantation, we found that OVA-induced IgE and IgG1 levels were reduced in OPN-deficient asthmatic mice, Th1/Th2 and Th17/Treg balance was maintained, gut barrier function was improved, and microbiome changes in OPN-deficient mice were compensated for, with an elevated abundance of *Allobaculum* and reduced abundance of *Desulfovibrio* and *Oscillibacter*. We further discovered that OPN deficiency reduces FOXP3 expression and decreases *Lactobacillus* colonization through activation of the PD-1/PD-L1 pathway in the intestine and lung. The present study suggests that OPN may moderate OVA-induced asthma by modulating the gut and lung microbiota.

**IMPORTANCE:**

Osteopontin deficiency exacerbated asthmatic airway inflammation, an effect associated with microbiota dysbiosis, impaired intestinal barrier function, and increased PD-1/PD-L1 expression and thus decreased Treg cell function. The study provides clinicians with new insights into asthma mechanisms and can also lead to new ideas for asthma treatment.

## INTRODUCTION

Asthma is a heterogeneous clinical syndrome that affects more than 300 million people worldwide (1). Allergic asthma is the most common asthma phenotype in up to 90% of the pediatric asthma population and up to 50% of the adult asthma population (2). Asthma prevalence is increasing every year, driven by urbanization and lifestyle changes (3). The pathogenesis of asthma remains incompletely elucidated and is associated with various genetic, environmental, infectious, and nutritional factors. Currently, traditional asthma treatments have certain limitations, as long-term use of steroids can lead to significant side effects and only provides temporary symptom relief rather than a curative solution (4–6). Therefore, there is an urgent need to further investigate the molecular mechanisms underlying asthma pathogenesis and to develop new therapeutic targets and treatment approaches.

Allergic asthma is defined by the presence of specific IgE sensitization to one or more aeroallergens (7). Allergen-specific CD4^+^ Th2 lymphocytes and their associated cytokines (interleukin-4 [IL-4], IL-5, and IL-13) are hallmark features of allergic asthma. Classical Th2 cytokines together with eosinophil chemokine-1 regulate key aspects of eosinophil recruitment, allergic inflammation, and airway hyperresponsiveness (AHR). Secretion of IL-4/IL-13 by Th2 cells facilitates class switching of B cells to produce allergy-specific IgE, which binds to FcεRI receptors on granulocytes, causing cross-linking of IgE upon allergen exposure, leading to degranulation and release of histamine, prostaglandins, and other inflammatory mediators. In addition, IL-5 secreted by Th2 cells promotes airway eosinophilia (8, 9).

Lung and gut microbiota play several important roles in the development, regulation, and maintenance of healthy immune responses. Imbalance between the symbiotic and pathological bacterial strains in the gut and lung in asthma may lead to altered immune development and inappropriate inflammatory responses (10). Patients with high Th2-related pulmonary inflammation exhibit lower bacterial diversity in their airways. Proteobacteria (such as *Haemophilus* and *Neisseria*) are enriched in asthma patients (11). *Haemophilus parainfluenzae* activates Toll-like receptor 4 (TLR4), which subsequently leads to the transcription of pro-inflammatory factors such as IL-8, while simultaneously inhibiting corticosteroid-related pathways (12).

The burgeoning field of gut-lung axis research has unveiled the intricate interplay between the gut microbiota and respiratory health, particularly in the context of asthma. Recent studies have demonstrated that dysbiosis of the gut microbiota can precipitate the onset and exacerbation of asthma. Germ-free mice exhibited an exaggerated susceptibility to allergic responses, indicating a pivotal role of commensal gut bacteria in regulating immune responses associated with asthma (13). Specific gut microbes, particularly probiotics including the genera *Lactobacillus* and *Bifidobacterium*, hold significant value in mitigating allergic asthma (14). *Clostridium* species have been demonstrated to increase the proportion of regulatory T cells (Tregs) in mice (15), and are negatively correlated with asthma (16). These findings suggest that gut microbiota species may regulate asthma by modulating airway inflammation. However, further studies are needed to more clearly define the dominant species involved and to understand whether bacterial dysbiosis in the context of asthma is the cause or effect of disease.

In allergic diseases, the anatomical and functional homeostatic balance of the epithelial barrier is skewed toward deleterious activation of the immune system, reduced junctional integrity, and impairment of epithelial barrier function. When the epithelial barrier is compromised, microorganisms, allergens, and other antigens can pass between epithelial cells through the basement membrane to the underlying tissue, triggering innate immune and adaptive immunity responses (17); therefore, more detailed mechanistic studies are necessary. Increased intestinal permeability due to exposure to antigens in asthmatics may play a role in susceptibility to environmental allergens. Correction of intestinal barrier defects may be another novel approach to asthma treatment.

Osteopontin (OPN) (encoded by the *Spp1* gene) is a soluble cytokine and a matrix-associated protein present in most tissues and body fluids (18). OPN promotes cytokine and inducible nitric oxide synthase (iNOS) expression, affecting phagocytosis and cell migration in macrophages (19), and participate in the generation, development, differentiation, and activation of dendritic cells (DCs) and regulate the expression of TLR9 and interferon α (IFN-α) (20). OPN also facilitates the maturation and differentiation of natural killer (NK) cells (21), enhances type 2 innate lymphoid cells (ILC2) proliferation, and upregulates the expression of GATA-3, RORα, IL-5, and IL-13 (22). Moreover, OPN increases the tendency of B cell (23) aggregation and suppresses T cell activation (24). Current research findings have confirmed that OPN demonstrates a high expression in asthma patients (25). In OPN-deficient mice, elevated levels of lung tissue injury markers and increased bacterial load in bronchoalveolar lavage fluid (BALF) and lung tissue are detected, suggesting that OPN plays a protective role in asthma (26). Additionally, OPN promotes antigenic tolerance in mediastinal lymph nodes by stimulating the production of IFN-β by plasmacytoid dendritic cells and enhancing Treg cell activity, preventing patients from allergic airway inflammation (27). Currently, research on OPN in asthma is limited, and its mechanisms of action remain incompletely understood, necessitating further investigation. Additionally, the role of the relationship between OPN and microbiota in asthma has yet to be elucidated.

OPN plays an important role in maintaining the integrity of the epithelial barrier. Research found that OPN maintains tight-junction (TJ) protein complexes, enabling occludin to localize to the TJs to be phosphorylated (28). OPN also has a role in regulating microbiota. Research found OPN modulates gut microbiota with enhanced health-associated commensal bacteria *Akkermansia* (29). In addition, the relationship between OPN and microorganisms has been reported in other diseases. A recent study showed that overexpression of OPN in intestinal epithelial cells (IECs) or administration of milk OPN maintains the intestinal microbiome by intestinal antimicrobial peptides. The increase in tryptophan metabolites and short-chain fatty acids signaling through the aryl hydrocarbon receptor in IECs preserves the intestinal barrier function and protects from alcohol-associated liver disease (30). Toyonaga et al. showed that OPN affects gut microbiota and macrophage phagocytic activity in the onset of spontaneous colitis (31).

Programmed cell death 1 (PD-1) and its ligand PD-L1 are receptors that act in co-stimulatory and coinhibitory immune responses (32). We recently found that PD-1 and PD-L1 are highly expressed in asthma and are associated with gut microbiota. However, how OPN regulates the microbiota and PD-1/PD-L1 pathway to improve asthma has not been investigated.

Thus, the aims of this work were, firstly, to compare the protective effects of wild-type mice versus OPN knockout mice on airway inflammation in asthma, secondly, to analyze if knockout of OPN could disorder the gut/lung microbiome and the intestinal epithelial barrier function to aggravate asthma, and thirdly, to assess whether the protective effect of OPN on asthma is mediated by the gut microbiota, thereby elucidating the association of protective effects of OPN with the microbiota and the mechanisms.

## MATERIALS AND METHODS

### Animals

Six- to 7-week-old male C57BL/6J mice (wild‐type or Spp1^+/+^ mice were used as controls and referred to as OVA in the text or figures for simplicity purposes only) were purchased from Fourth Military Medical University. Six- to 7-week-old male C57BL/6J OPN knockout mice (Spp1^−/−^) were purchased from The Jackson Laboratory (Bar Harbor, ME); the Spp1^−/−^ mice have been described elsewhere (33). All mice were housed in Individually ventilated caging (IVC) individually ventilated cages with no more than five mice per cage at a temperature of 22 ± 1°C, at a humidity of 50 ± 10%, and with a 12 hour light-dark cycle. Mice were free to access food and water. All mice were acclimatized for 1 week before the start of the experiment, and the acclimatization conditions were consistent with the experimental period.

### Model of OVA-induced airway inflammation and experimental design

**Fig 1:**
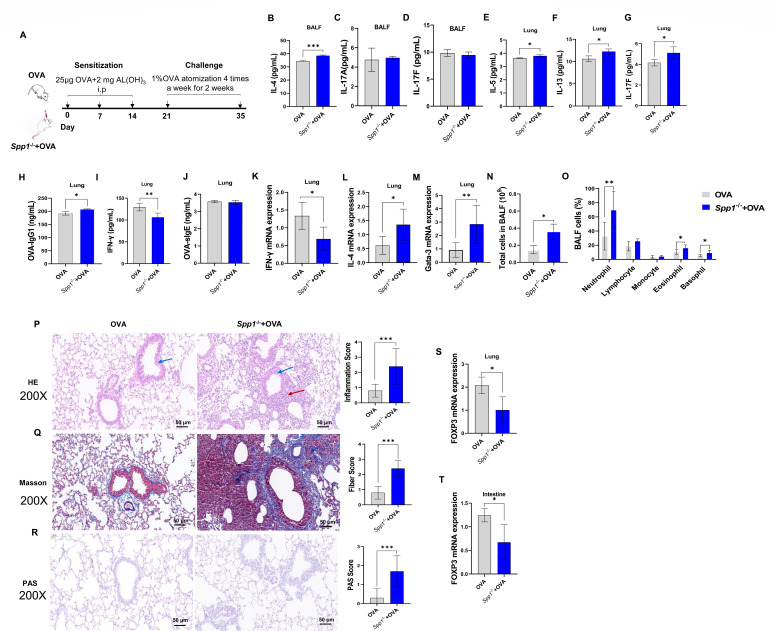
OPN knockout mice display increased inflammatory cell recruitment and inflammatory cytokines, increased tissue damage, and Th2 cell polarization during allergic airway inflammation. (**A**) Schematic presentation of the experimental design. (**B**) Levels of IL-4 in BALF. (**C and D**) Levels of IL-17A/F in BALF. (**E–J**) Levels of inflammatory cytokines in the lung. (**K–M**) Th1 and Th2 polarization in the lung. (**N**) Total leukocytes in BALF. (**O**) Differential cell counts in BALF. (**P**) Lung tissue was stained with hematoxylin and eosin (HE) to detect lung damage. (**Q**) Masson staining of lung tissue was used to determine pulmonary fibrosis. (**R**) PAS staining of lung tissue was used to determine pulmonary mucus secretion. (**S**) FOXP3 expression in the lung. (**T**) Expression of FOXP3 in small intestine. Enzyme-linked immunosorbent assay was performed to detect IL-4, IL-17A/F in BALF, and inflammatory factors in the lungs. qPCR was performed to detect the expression of Th1/Th2 polarization and FOXP3. Total leukocytes and differential cells in BALF were determined by an automated cell counter. The data are the means ± SD; *P < 0.05, **P < 0.01, ***P < 0.001. OVA-IgG1, OVA-specific immunoglobulin G1; GATA-3, Gata binding protein 3; FOXP3, forkhead box P3.

The *in vivo* experiments consist of two parts (Fig. 1A and 5A). For part 1, to confirm the role of OPN in asthma, mice were stochastically divided into two groups (10 mice per group): the OVA group and Spp1^−/−^+OVA group. An asthma model was generated using sensitization and challenge with OVA. All groups were sensitized by intraperitoneal injection of 25 µg OVA/2  mg Al(OH)_3_ gel in 0.2 mL phosphate-buffered saline (PBS) on days 0, 7, and 14. After the last time of OVA injection, the mice were then challenged by intranasal inhalations with 5% OVA for 30 min/day for 2 weeks (once every other day). On the final day of treatment, mice were euthanized under anesthesia. Lung, ileum tissue, and BALF were harvested for histopathology and evaluation of the development of allergic airways.

For part 2, after confirming the protective effect of OPN on asthma, we performed fecal microbiota transplantation (FMT) on Spp1^−/−^ mice to elucidate whether the protective effect of OPN on asthma was related to the microbiota. In brief, 20 mice were randomly divided into two groups: the Spp1^−/−^+OVA group and the Spp1^−/−^+OVA+FMT group. The sensitization and challenge protocols were identical to those described in part 1. During the challenge, the Spp1^−/−^+OVA+FMT group was given fecal microbiota, and the Spp1^−/−^+OVA group was given PBS. Finally, mice were executed to euthanasia. Then, the BALF was collected for the next research. In addition, a part of lung and ileum tissues were fixed in 4% formaldehyde, and the remaining lung tissues were stored at −80°C for further experiments.

### FMT experiment

FMT was performed according to the modified method described previously (34). Briefly, fresh (donor mice) feces from normal mice were collected and resuspended in 200 mg/1 mL of PBS, and then the dissolved feces were centrifuged at 1,000 × *g* (4°C) for 3 min. The suspension was collected, and 0.2 mL of bacterial suspension was gavaged to the Spp1^−/−^+OVA+FMT mice (receptor mice). It was ensured that the gavage was finished within 10 min. All mice had free access to food and water and were gavaged in an specific pathogen-free (SPF) environment. After the mice were transplanted for 2 weeks, specimens of BALF, ileum, lungs, and other specimens were taken for further examination.

### Cell counting in BALF

Immediately after euthanizing the mice, BALF was collected. Specific operations are as follows: their abdomens were fixed upward on the experimental work table, and their tracheas were exposed anatomically and intubated. When BALF was collected, a pre-cooled sterile PBS solution of 0.6 mL was aspirated with 1 mL of a disposable sterile syringe, connected to a fixed endotracheal tube, and the pre-cooled PBS solution was slowly injected into the lungs of mice. After the solution injection was completed, there was a 1 min dwell before withdrawal, repeated three times. Collected BALF was centrifuged at 500 × *g* for 10 min at 4°C. incell countsThe cell pellet was then resuspended in PBS for white blood cell counts using an automated cell counter (XPEN55 CRP&SAA, Shenzhen XPENARRAY Biotechnology Co., Ltd., Shenzhen, China), and the supernatant was collected for cytokine level analysis.

### Airway hyperresponsiveness tests

Twenty-four hours after the last challenge, mice were weighed and anesthetized with an intraperitoneal injection of 2% sodium pentobarbital (50 mg/kg, Sigma-Aldrich, St. Louis, MO, USA). Mice underwent tracheostomy under anesthesia and were intubated with a tracheal cannula. The proximal end of the cannula was connected to a Y-tube adapter from a computer-controlled small animal ventilator system (FlexiVent, EMKA, France) for mechanical ventilation. First, normal saline was added to the atomizer. After nebulization, resistance (Rrs) values were recorded in each group. Then, inhalation of 1.5, 3, 6, 12, 25, 50, 100 mg/mL of methacholine (Mch, Sigma-Aldrich) was followed by 2 min of normal ventilation; the Rrs values for each group of mice under corresponding Mch stimulation were recorded for airway hyperresponsiveness assessment.

### Enzyme-linked immunosorbent assay (ELISA)

The IL-4, OVA-sIgE, OVA-IgG1, and IL-10 levels in the supernatant of BALF and lung tissues and IL-5, IL-13, IFN-γ, IL-17F, matrix metallopeptidase-9 (MMP9), transforming growth factor-β (TGF-β), and vascular endothelial growth factor (VEGF) in lung tissues were determined by ELISA Kits (Jiangsu Enzyme Label Biotechnology Co., Ltd., Jiangsu, China). The experimental procedures followed the instructions.

### Real-time quantitative PCR analysis

RNA was extracted from the lung and small intestine tissues by using the total RNA Kit, and reverse transcription was performed to gain cDNA with the SmArt reverse transcriptase (Thermo Fisher Scientific, Inc., USA) according to the manufacturer’s instructions. Quantitative real-time PCR was performed using Power SYBR PCR Master Mix (Applied Biosystems) and a two-step PCR program (95°C for 5 s, 60°C for 60 s, 40 cycles). The data were analyzed using the comparative 2^−ΔΔCt^ method and normalized to β-actin mRNA as an internal control. Primer sequences employed are summarized in Table 1.

**TABLE 1:** Primer sequences for real-time PCR

Gene	Forward primer (5′ to 3′)	Reverse primer (5′ to 3′)
IL-4	ACAGGAGAAGGGACGCCAT	GAAGCCCTACAGACGAGCTCA
GATA-3	GCCTGTECAAAAGAGATTTCAGAT	TGATTCACAGAGCATGTAGECC
IFN-γ	TGGCCTCCCTCTCATCAGTT	TTGAGATCCATGCCGTTGGC
ZO-1^*a*^	GAGCGGGCTACCTTACTGAAC	GTCATCTCTTTCCGAGGCATTAG
Claudin	GAACAGACTACAGGCACTT	TGGACATTAAGGCAGCAT
Occludin	TGAAAGTCCACCTCCTTACAGA	CCGGATAAAAAGAGTACGCTGG
PD-1	GTCCCTCACCTTCTACCC	GGTTCCAGTTCACATAAGA
PD-L1	TATCACEECTCCAAAGGACT	ACCACTAACECAAGCAGGTC
β-Actin	GGCTGTATTCCCCTCCATCG	CCAGTTGGTAACAATGCCATGT

^*a*^ ZO-1, zonula occludens-1.

### Histology and immunohistochemistry

Lung and ileum tissue was freshly collected and fixed with 10% neutral buffered formalin, and cut longitudinally into 5 µm sections following paraffin embedding to hematoxylin and eosin (H&E) or periodic acid-Schiff (PAS) or Masson staining. All histological assessments were performed by two independent pathologists in a blinded manner. The inflammation score was performed according to previously established criteria (35) with the following grading scale: grade 0 (normal), grade 1 (inflammatory cells were occasionally seen), grade 2 (inflammatory cells surrounded 1–3 layers of bronchi), grade 3 (inflammatory cells surrounded 4–5 layers of bronchi), and grade 4 (most inflammatory cells surrounded more than five layers of bronchi). PAS scoring referenced previous study (36): 0 indicates a negative result, while 1–4 is positive. The amount of mucus production in positively stained bronchi was rated according to the following criteria: 1, 5%–25%; 2, 25%–50%; 3, 50%–75%; and 4, >75%. The severity of pulmonary fibrosis was evaluated using a modified scoring system based on previous studies (37), with the following criteria: 0, normal tissues devoid of alveolitis or fibrosis; 1, mild alveolitis or fibrosis with ≤20% lung lesions; 2, moderate alveolitis or fibrosis with 20%–50% lung lesions; 3, severe alveolitis or fibrosis with ≥50% lung lesions.

Paraffin sections of lung and ileum were deparaffinized in xylene and hydrated through graded alcohols, incubated in 3% hydrogen peroxide solution, and blocked in 3% bovine serum albumin. Sections were then incubated overnight at 4°C with primary antibodies against PD-1 and PD-L1, respectively, followed by horseradish peroxidase (HRP) enzyme-labeled goat anti-rabbit secondary antibodies and goat anti-mouse secondary antibodies (Proteintech Group, Inc., Wuhan, China), incubation for 1 h at 37°C. Immunoreactivity was visualized using diaminobenzidine chromogen substrate, followed by hematoxylin counterstaining. Sections were then dehydrated through graded alcohols, sealed, and the results were read. Under the white light microscope, the positive areas exhibited a brown or brownish-yellow appearance. The average optical density was calculated using ImageJ (38).

**Fig 2:**
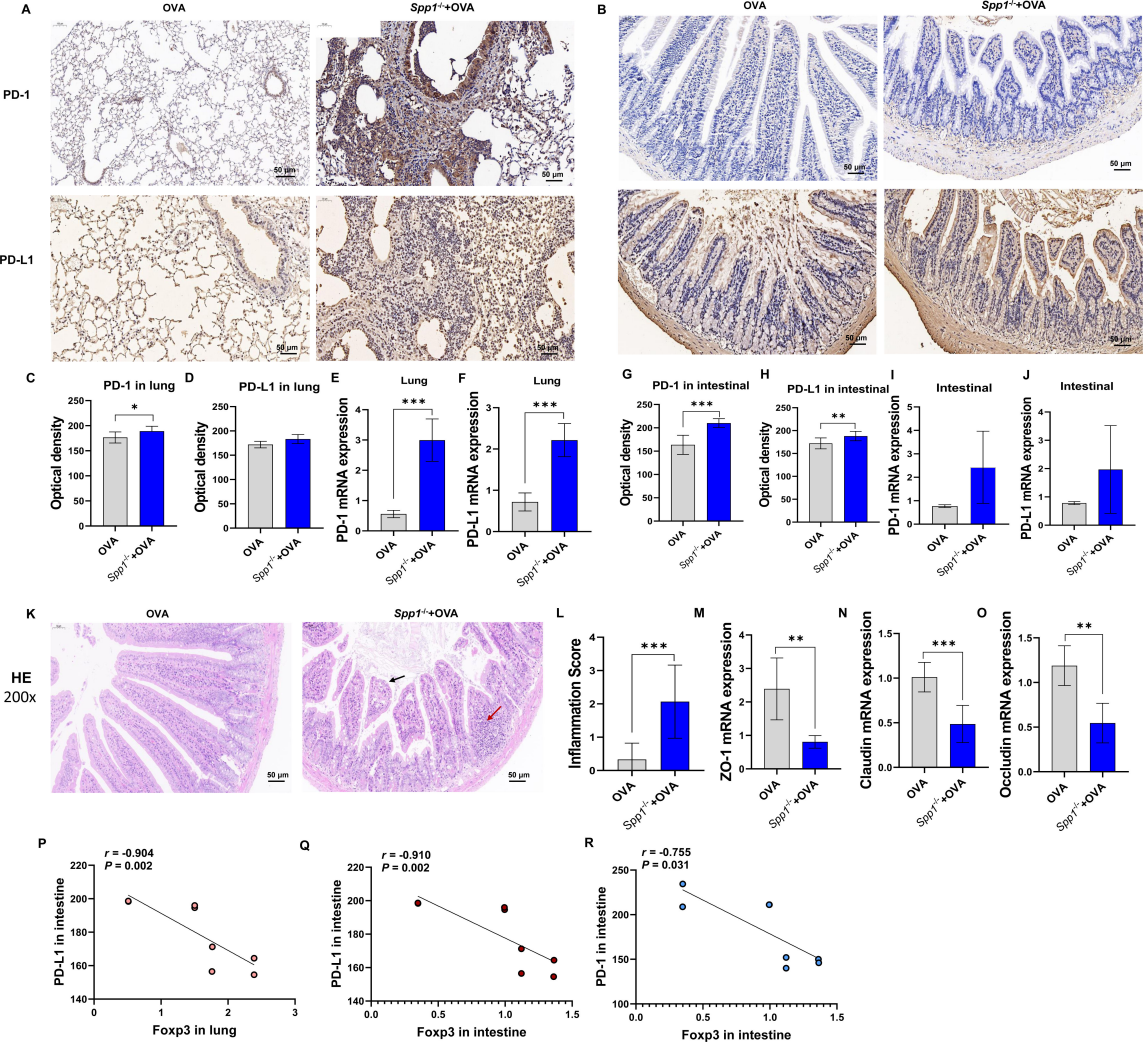
OPN knockout affects Treg cell accumulation and expression of PD-1 and PD-L1 and displays increased gut permeability. (**A and B**) Immunohistochemistry examination of PD-1 and PD-L1 in the lung and small intestine (200×). (**C**) Optical density of PD-1 in the lung. (**D**) Optical density of PD-L1 in the lung. (**E**) PD-1 mRNA expression in the lung. (**F**) PD-L1 mRNA expression in the lung by qPCR. (**G**) Optical density of PD-1 in the small intestine. (**H**) Optical density of PD-L1 in the small intestine. (**I and J**) PD-1 and PD-L1 mRNA expression in the small intestine by qPCR. (**K**) HE staining of small intestine (200×). (**L**) Small intestinal inflammation score. (**M–O**) ZO-1, claudin, and occludin mRNA expression in the small intestine by qPCR. (P–R) The association of FOXP3 expression in both intestinal and lung tissues with PD-1/PD-L1 pathway in the intestine. The data are the means ± SD; *P < 0.05, **P < 0.01, ***P < 0.001. ZO-1, zonula occludens-1; HE, hematoxylin and eosin.

In certain cases, the same images are used to present different analytical results. For example, the Spp1^−/−^+OVA group images in Fig. 1P through R and 5L through N and the Spp1^−/−^+OVA group images in Fig. 2A and K and 6A, E, and F use the same original images but are employed to demonstrate the pathological changes in tissues after Spp1 knockout and the improvement in tissues after FMT, respectively. This reuse is intended to ensure consistency in analysis and to avoid bias caused by sample differences. Additionally, all reused images are clearly indicated in the relevant figure legends.

### Sample DNA isolation and 16S RNA sequencing

Fresh fecal pellets and BALF were collected and immediately stored at −80°C until use. BALF DNA was extracted using the Mabio Bacterial DNA Extraction Mini Kit, and fecal DNA was extracted using the DC306-09 Magnetic Bead Soil DNA Kit following the improved protocol based on the manufacturer’s instructions. The purity and concentration of the DNA are assessed using a Nanodrop One (Thermo Fisher Scientific, MA, USA). Using genomic DNA as the template, PCR amplification is performed according to the selected sequencing regions, employing specific primers bearing barcodes and Premix Taq. The V3-V4 regions of the microbial 16S RNA were amplified with the paired primers (forward primer: 5′-CCTACGGGRSGCAGCAG-3′; reverse primer: 5′-GGACTACVVGGGTATCTAATC-3′). PCR conditions were 94°C for 5 min, followed by 30 cycles at 94°C for 30 s, then 52°C for 30 s, and 72°C for 30 s, and a final extension at 72°C for 10 min. Electrophoresis of PCR products is conducted on a 1% agarose gel to assess the fragment length and concentration. Samples with main bands falling within the normal range of 290 bp–310 bp are suitable for further experimentation. Following the concentration comparison of PCR products using GeneTools Analysis Software (version 4.03.05.0, SynGene), the required volume for each sample is calculated based on the principle of equal mass, after which the PCR products are mixed. The PCR mixture is then recovered using the E.Z.N.A. Gel Extraction Kit (Omega, USA), with target DNA fragments eluted in Tris-EDTA (TE) buffer. Library construction is carried out following the standard protocol of the ALFA-SEQ DNA Library Prep Kit, and the size of the library fragments is evaluated on the Qsep400 High-Throughput Nucleic Acid & Protein Analysis System (Hangzhou Houze Biotechnology Co., Ltd., China). The concentration of the library is measured using a Qubit 4.0 (Thermo Fisher Scientific, Waltham, MA, USA). The constructed amplicon libraries are subjected to PE250 sequencing on either the Illumina or MGI platform (Guangdong Magigene Biotechnology Co., Ltd., Guangzhou, China). After removing chimeric sequences, operational taxonomic units (OTUs) were classified based on the 97% similarity of valid sequences across all samples. Principal coordinate analysis (PCoA) and principal component analysis (PCA) were used to investigate variations in the gut microbiota composition between groups and samples. The Shannon and Simpson indexes were used to measure sample diversity. Using linear discriminant analysis and the effect size analysis method pinpointed the primary contributing bacteria in charge of sample variations. Microbiome composition was analyzed at both phylum and genus taxonomic levels.

### Statistical analysis

All analyses were performed using GraphPad Prism software (version 8.0). The data are shown as mean ± standard deviation (SD). Statistical analyses were performed using two-sided Student’s *t*-tests or by one-way analysis of variance. Non-parametric analysis was carried out using the Mann-Whitney U-test and the Wilcoxon signed-rank test. To obtain the correlations between different experiments, Spearman correlation analysis was performed by R (v.3.5.1). *P*-values <0.05 were considered statistically significant.

## RESULTS

### OPN knockout increased allergic inflammation

Comparative analysis of BALF cytokine levels revealed distinct inflammatory patterns between Spp1^−/−^+OVA and OVA-challenged mice. ELISA demonstrated significantly elevated IL-4 levels in Spp1^−/−^+OVA mice compared to OVA mice (Fig. 1B), in contrast, no statistically significant differences were observed for Th17-associated cytokines IL-17A (Fig. 1C) or IL-17F (Fig. 1D) between groups. When comparing Spp1^−/−^+OVA mice to OVA mice, lung tissues showed considerably higher levels of IL-5 (Fig. 1E), IL-13 (Fig. 1F), IL-17F (Fig. 1G), and OVA-specific IgG1 (Fig. 1H) and significantly lower levels of IFN-γ (Fig. 1I). But changes in IgE were not significant. In the lung (Fig. 1J), Th1 polarization (Fig. 1K) was significantly inhibited, and Th2 polarization (Fig. 1L and M) was significantly promoted in the Spp1^−/−^+OVA group compared with the OVA group. Additionally, we discovered that following OPN knockout, the overall number of leukocytes in BALF (Fig. 1N)—including neutrophils, eosinophils, and basophils (Fig. 1O)—was markedly elevated. However, we observed no discernible variation in airway hyperresponsiveness (Fig. S1A) or airway remodeling (Fig. S1B) of Spp1^−/−^ mice. These results imply that OPN knockout has a detrimental effect on airway inflammation in asthmatic mice.

### Forkhead box P3 (FOXP3) expression was reduced, and tissue damage increased during allergic inflammation in OPN knockout mice

To investigate whether OPN affects Treg cell function and lung tissue damage, wild-type and Spp1^−/−^ mice were subjected to the i.p. injection and atomized inhalation of OVA. Thereafter, lung tissue was harvested. Lung histopathological changes were assessed by H&E, PAS, and Masson staining, while FOXP3 mRNA expression was quantified via real-time quantitative PCR. H&E staining revealed a significant increase in inflammatory infiltration (indicated by the red arrows) in lung tissue and a smaller airway compartment (indicated by the blue arrows) in lung tissue of Spp1^−/−^+OVA mice compared to OVA mice (Fig. 1P); Masson staining revealed significant fibrosis in the lung tissue of Spp1^−/−^+OVA mice, consistent with a significant increase in fiber scores (Fig. 1Q); and PAS staining revealed increased mucus secretion in the lung tissue of Spp1^−/−^+OVA mice and a significant increase in PAS scores (Fig. 1R). Furthermore, we found a significant decrease in FOXP3 expression (Fig. 1S and T) in the lung and small intestine tissues of Spp1^−/−^+OVA mice.

### OPN knockout increased the expression of PD-1/PD-L1

To clarify how OPN affects asthma, we analyzed levels of PD-1 and PD-L1 in the lungs and small intestine. As shown in Fig. 2, immunohistochemistry showed that PD-1/PD-L1 total positive cell counts in the lung and small intestine were significantly increased in Spp1^−/−^+OVA mice compared with OVA mice (Fig. 2A and B). Optical density quantification confirmed enhanced PD-1/PD-L1 protein levels in Spp1^−/−^+OVA group in the lung and small intestine (Fig. 2C, D, G, and H) compared with OVA group. We then detected the mRNA expression levels of PD-1 and PD-L1 in the lungs and small intestine using real-time fluorogenic quantitative PCR and found that the mRNA expression levels of PD-1 and PD-L1 were increased in Spp1^−/−^+OVA mice compared with OVA mice (Fig. 2E, F, I, and J). Moreover, the expression of FOXP3 was significantly correlated with the levels of PD-1 and PD-L1 in the intestine (Fig. 2P through R). These results suggest that OPN knockout aggravates asthma by increasing the expression of PD-1 and PD-L1 and thereby decreasing FOXP3 expression.

### OPN knockout aggravates gut permeability

Intestinal barrier dysfunction contributes to the pathogenesis of allergic asthma (39). We measured gut permeability in wild‐type and Spp1^−/−^ mice. By H&E staining of the small intestine, we found that compared to OVA mice, Spp1^−/−^+OVA mice had broken small intestinal villi (shown by black arrows) and a large infiltration of inflammatory cells (shown by red arrows, Fig. 2K), and the inflammation score of Spp1^−/−^+OVA mice was also significantly increased (Fig. 2L). Furthermore, we examined the expression of proteins related to intestinal barrier function and found that zonula occludens-1 (ZO-1), claudin, and occludin were significantly decreased in the small intestine of Spp1^−/−^+OVA mice compared with that of OVA mice (Fig. 2M through O). These results suggest that the OPN knockout breaks down the intestinal epithelial barrier function in asthmatic mice.

### OPN knockout aggravates OVA-induced microbiome dysbiosis

Microbial imbalance in asthma compromises intestinal barrier integrity. When the barrier is impaired, microorganisms, allergens, and other antigens can pass between epithelial cells through the basement membrane to the underlying tissue, triggering aberrant immune responses. Furthermore, bacterial metabolites from the gut exert an influence on the lungs either via the circulation or through migration of immune cells stimulated by bacterial factors (40). Given this gut-lung axis effect, we systematically evaluated whether OPN confers protection against respiratory and gut microbiota dysbiosis in asthmatic mice by performing 16S rRNA sequencing of fecal and BALF samples.

#### The microbiome in BALF

**Fig 3:**
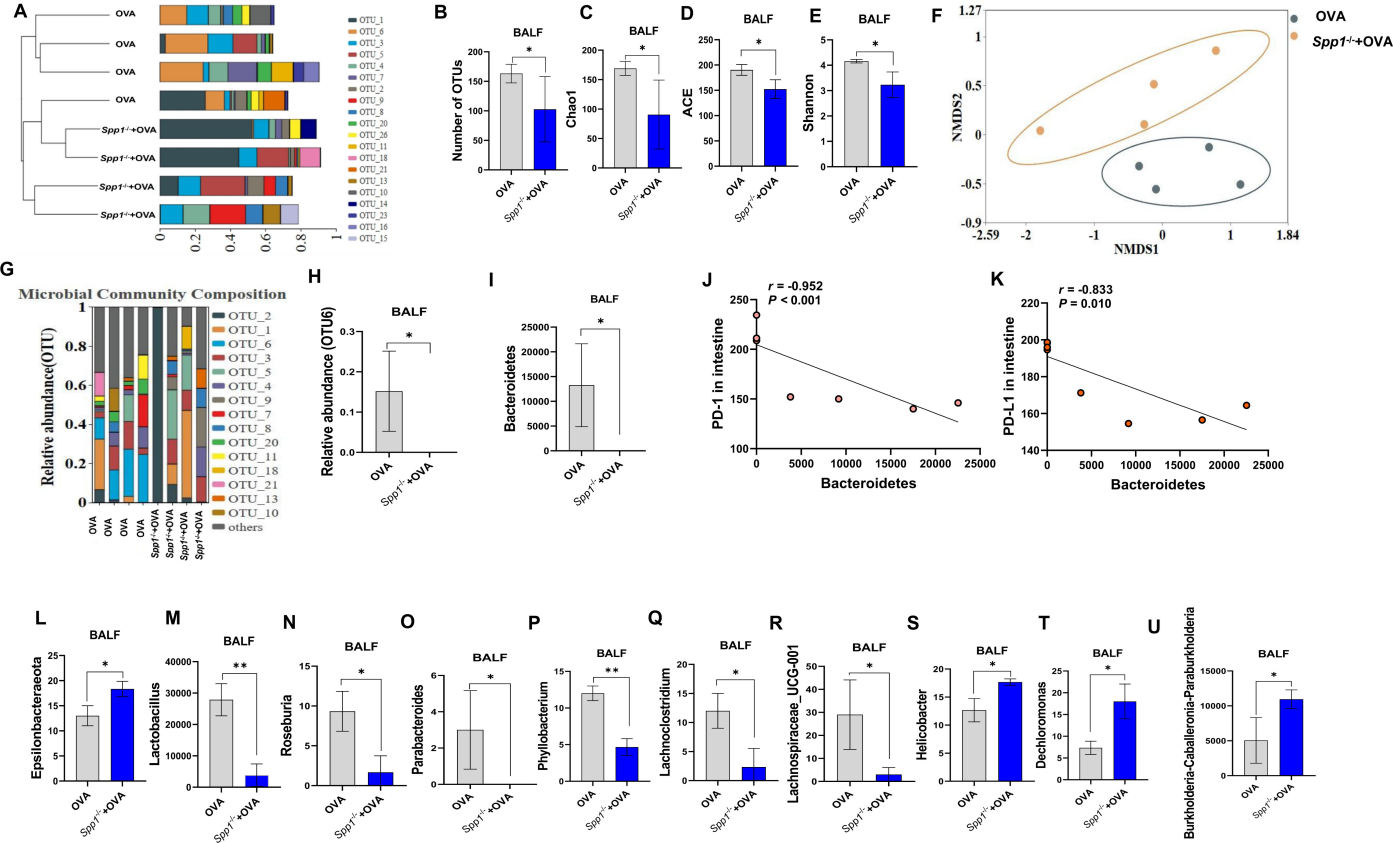
OPN prevents microbiome dysbiosis in BALF. (**A**) Demonstrate the similarity between samples in the form of a hierarchical tree through the analytical method of hierarchical clustering. (**B**) Number of OTUs included in each group. (**C–E**) Microbial diversity (Chao1, ACE, Shannon, and Simpson index diversity indicators) was assessed by Kruskal-Wallis test. (**F**) Beta diversity (Bray-Curtis dissimilarity index) visualized by NMDS. (**G and H**) Composition of microbiota at OTU and genus levels. (**I**) *Bacteroidetes* at phylum levels (Wilcoxon test). (**J and K**) Correlation between *Bacteroidetes* and PD-1/PD-L1. (**M–U**) Genus-level bacteria (Wilcoxon test). *P < 0.05. NMDS, non-metric multidimensional scaling; L, lung; In, intestine; Inf, INF-γ.

Using hierarchical clustering methods, we found that there were differences in the microbiome composition of Spp1^−/−^+OVA and OVA mice (Fig. 3A). Abundance of total bacteria was significantly reduced in Spp1^−/−^+OVA mice compared to OVA mice (Fig. 3B). The Shannon diversity, Chao1, and ACE were lower in Spp1^−/−^+OVA mice than in OVA mice, indicating a decreased diversity and richness microbiome in Spp1^−/−^+OVA mice (Fig. 3C through E). Weighted UniFrac analysis revealed that the composition and structure of the BALF bacteria in Spp1^−/−^+OVA mice were different from OVA mice (Fig. 3F). Microbial community composition analysis showed that Spp1^−/−^+OVA mice differed from OVA mice in their microbiome composition and relative abundance at the OTU (Fig. 3G) and genus levels (Fig. S2A); in particular, OTU6 was lacking after OPN knockout (Fig. 3H). At the phylum and genus level, we calculated the abundances of representative dominant bacteria. *Bacteroidetes* at the phylum level were significantly reduced (Fig. 3I). Spearman correlation analysis showed a significant negative correlation between the relative abundance of *Bacteroidetes* and PD-1/PD-L1 in the intestine (Fig. 3J and K). *Epsilonbacteraeota* was significantly increased in Spp1^−/−^+OVA mice compared to OVA mice (Fig. 3L). Differential abundance analysis at the bacterial genus level revealed nine significantly altered taxa between Spp1^−/−^+OVA and OVA mice (Fig. 3M through U): *Lactobacillus*, *Roseburia*, *Parabacteroides*, *Phyllobacterium*, *Lachnoclostridium*, and *Lachnospiraceae_UCG-001* were significantly decreased in Spp1^−/−^+OVA mice compared with OVA mice, and *Helicobacter*, *Dechloromonas*, and *Burkholderia-Caballeronia-Paraburkholderia* were significantly increased in Spp1^−/−^+OVA mice. The Spearman correlation showed a positive correlation between claudin and *Lactobacillus* and a negative correlation between IL-4, IL-17F in the lung, expression of PD-L1 in the small intestine, and *Lactobacillus*, as well as the abundances of *Burkholderia-Caballeronia-Paraburkholderia* positively correlated with GATA-3 in the lung (Fig. S2B).

#### Analysis of the feces microbiota found

**Fig 4:**
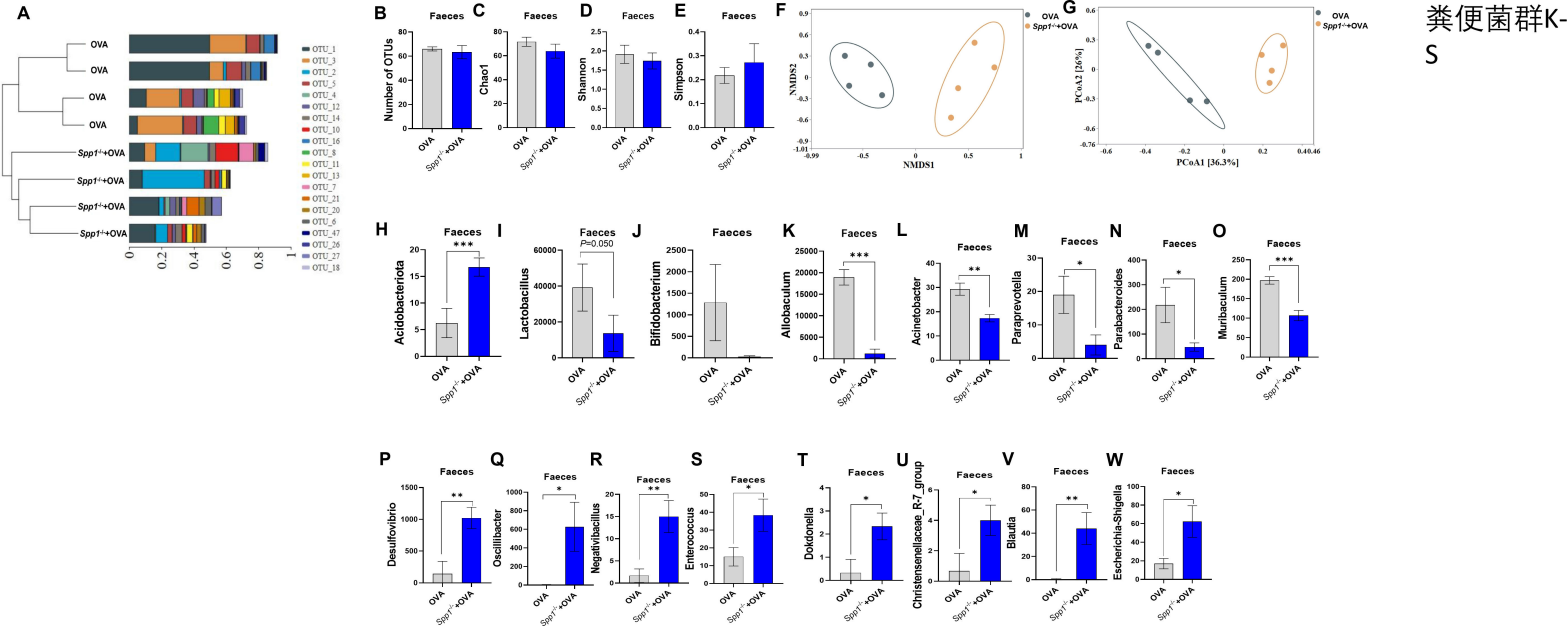
OPN prevents microbiome dysbiosis in feces. (**A**) Demonstrate the similarity between samples in the form of a hierarchical tree through the analytical method of hierarchical clustering. (**B**) Number of OTUs included in each group. (**C–E**) Microbial diversity (Chao1, Shannon, and Simpson index diversity indicators) was assessed by Kruskal-Wallis test. (**F–G**) Beta diversity (Bray-Curtis dissimilarity index) visualized by NMDS and PCoA. (**H**) Related abundance of bacteria at phylum level (**I–W**) and genus levels. *P < 0.05, **P < 0.01; ***P < 0.001. NMDS, non-metric multidimensional scaling; L, lung; In, intestine; Inf, INF-γ.

Cluster analysis showed that the microbiome composition was different in Spp1^−/−^+OVA compared with OVA mice (Fig. 4A). Alpha diversity showed that the number of OTUs (Fig. 4B), Chao1 (Fig. 4C), and Shannon (Fig. 4D) were lower in Spp1^−/−^+OVA mice than in OVA mice and Simpson was higher in Spp1^−/−^+OVA mice than OVA mice; there was no significant difference (Fig. 4E). Beta diversity revealed that the composition and structure of the fecal microbiome in Spp1^−/−^+OVA mice was different from OVA mice (Fig. 4F and G). At the phylum level, *Acidobacteriota* was significantly increased in Spp1^−/−^+OVA mice compared to OVA mice (Fig. 4H). Microbial community composition analysis showed that Spp1^−/−^+OVA mice differed from OVA mice in their microbiome composition and relative abundance at genus levels (Fig. S3A). Compared with OVA mice, the relative abundance of *Lactobacillus* and *Bifidobacterium* decreased in Spp1^−/−^+OVA mice, but there was no significant difference (Fig. 4I and J). The relative abundance of *Allobaculum*, *Acinetobacter*, *Paraprevotella*, *Parabacteroides*, and *Muribaculum* significantly decreased (Fig. 4K through O). The relative abundance of *Desulfovibrio*, *Oscillibacter*, *Negativibacillus*, *Enterococcus*, *Dokdonella*, *Christensenellaceae_R-7_group*, *Blautia*, and *Escherichia-Shigella* was significantly increased in Spp1^−/−^+OVA mice compared to OVA mice (Fig. 4P through W). Spearman's correlation analysis showed that *Allobaculum* was significant positive correlation with FOXP3 and IFN-γ in the lungs and a significant negative correlation with IL-4 levels in the BALF, GATA-3 expression in the lungs, and PD-1 expression in the small intestine (Fig. S3B). Overall, these findings suggest that OPN knockout mice are unprotected from OVA-induced microbiome dysbiosis.

### Anti-inflammation activity of OPN is microbiome-dependent

**Fig 5:**
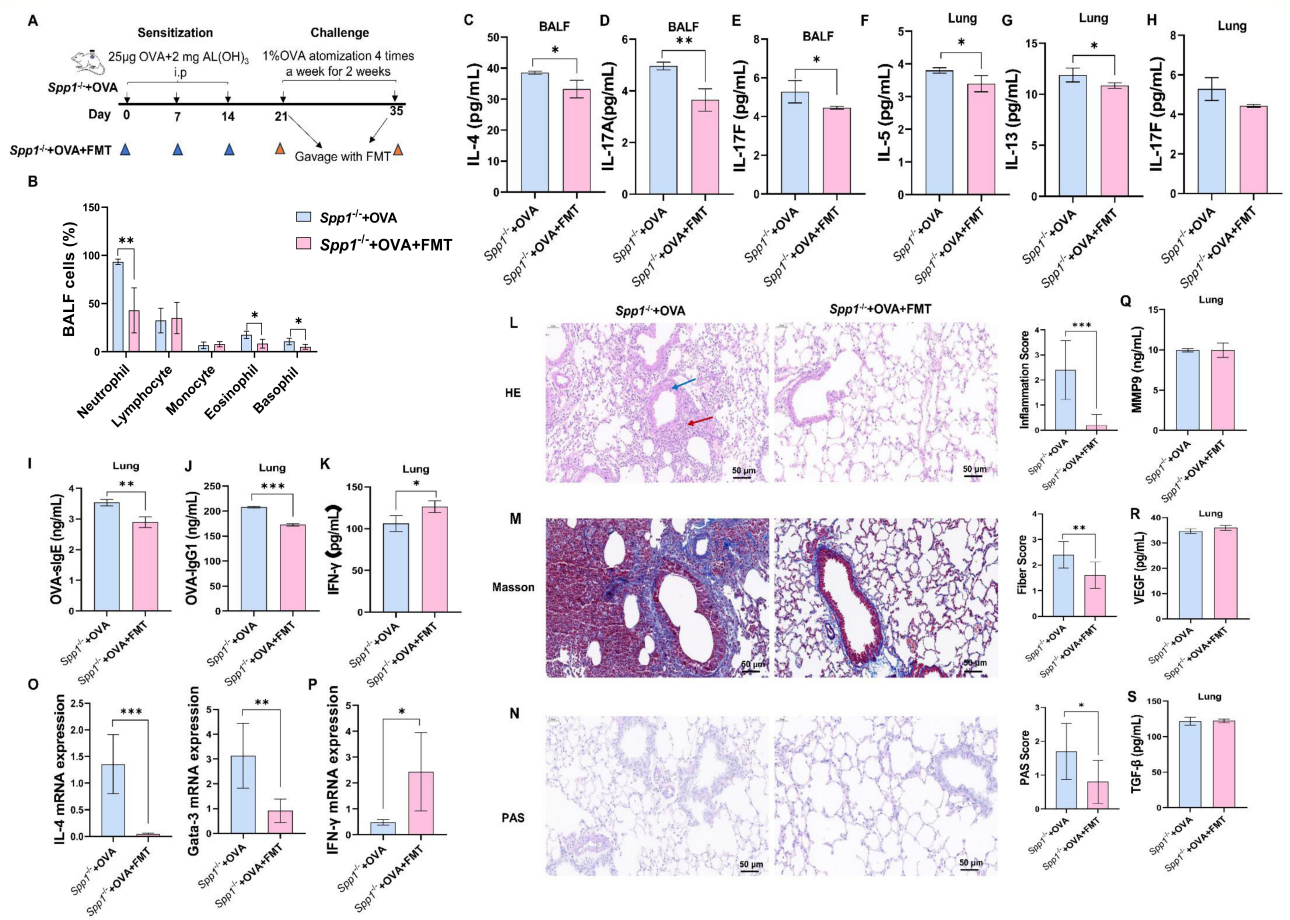
FMT protects from asthma in OPN knockout asthma mice. (**A**) Experimental design of FMT studies. (**B**) Total cells in BALF. (**C–E**) Concentrations of IL-4, IL-17A, and IL-17F in BALF. (**F–K**) Concentrations of IL-5, IL-13, IL-17F, OVA-specific IgE, OVA-specific IgG1, and IFN-γ in lung tissue. (**L**) Hematoxylin and eosin (HE) staining (200×) and inflammation score of lung tissue. (**M**) Masson staining (200×) and fiber score of lung tissue. (**N**) PAS staining (200×) and PAS score of lung tissue. (**O and P**) Th1 and Th2 polarization in the lung. (**Q–S**) Concentrations of MMP9, VEGF, and TGF-β in lung tissue. The data are the means ± SD; *P < 0.05, **P < 0.01, ***P < 0.001. ELISA was performed to detect inflammatory factors and MMP9, VEGF, and TGF-β in the BALF/lung. qPCR was performed to detect the expression of Th1/Th2 polarization. Differential cells in BALF were determined by an automated cell counter. Figure 1P through R (Spp1^−/−^+OVA group) and panels L through N (Spp1^−/−^+OVA group) use the same images to present different analytical perspectives. OVA-sIgE and OVA-IgG1, OVA-specific immunoglobulin E and immunoglobulin G.

To determine if microbiome protects asthma in OPN knockout mice, we transplanted fecal microbiome (FMT, from normal mice) to Spp1^−/−^ mice for 2 weeks. Neutrophil, eosinophil, and basophil counts were significantly lower in the BALF of Spp1^−/−^ mice that received FMT (Spp1^−/−^+OVA+FMT group) compared to mice that did not receive FMT (Spp1^−/−^+OVA group, Fig. 5B). Compared with the Spp1^−/−^+OVA group, IL-4, IL-17A, and IL-17F in BALF (Fig. 5C through E) and IL-5, IL-13, IL-17F, OVA-specific IgE and IgG1 in lung tissues (Fig. 5F through L) were significantly lower in the Spp1^−/−^+OVA+FMT group; IFN-γ levels in lung tissues were significantly higher (Fig. 5K). H&E staining (Fig. 5L), the inflammation scores, Masson staining, fiber score (Fig. 5M), PAS staining, and PAS score (Fig. 5N) showed less inflammatory infiltration, tracheal stenosis, fibrosis, and mucus formation in Spp1^−/−^+OVA+FMT group mice compared with Spp1^−/−^+OVA mice. Th2 polarization was significantly inhibited (IL-4, GATA-3, Fig. 5O), and Th1 polarization (IFN-γ, Fig. 5P) was significantly promoted in the Spp1^−/−^+OVA+FMT group compared with the Spp1^−/−^+OVA group. There was no significant difference in airway remodeling between the two groups (Fig. 5Q through S). The above results illustrate that FMT attenuates allergic inflammation and tissue damage in OPN knockout mice.

### FMT maintains intestinal barrier function in OPN knockout asthma mice

**Fig 6:**
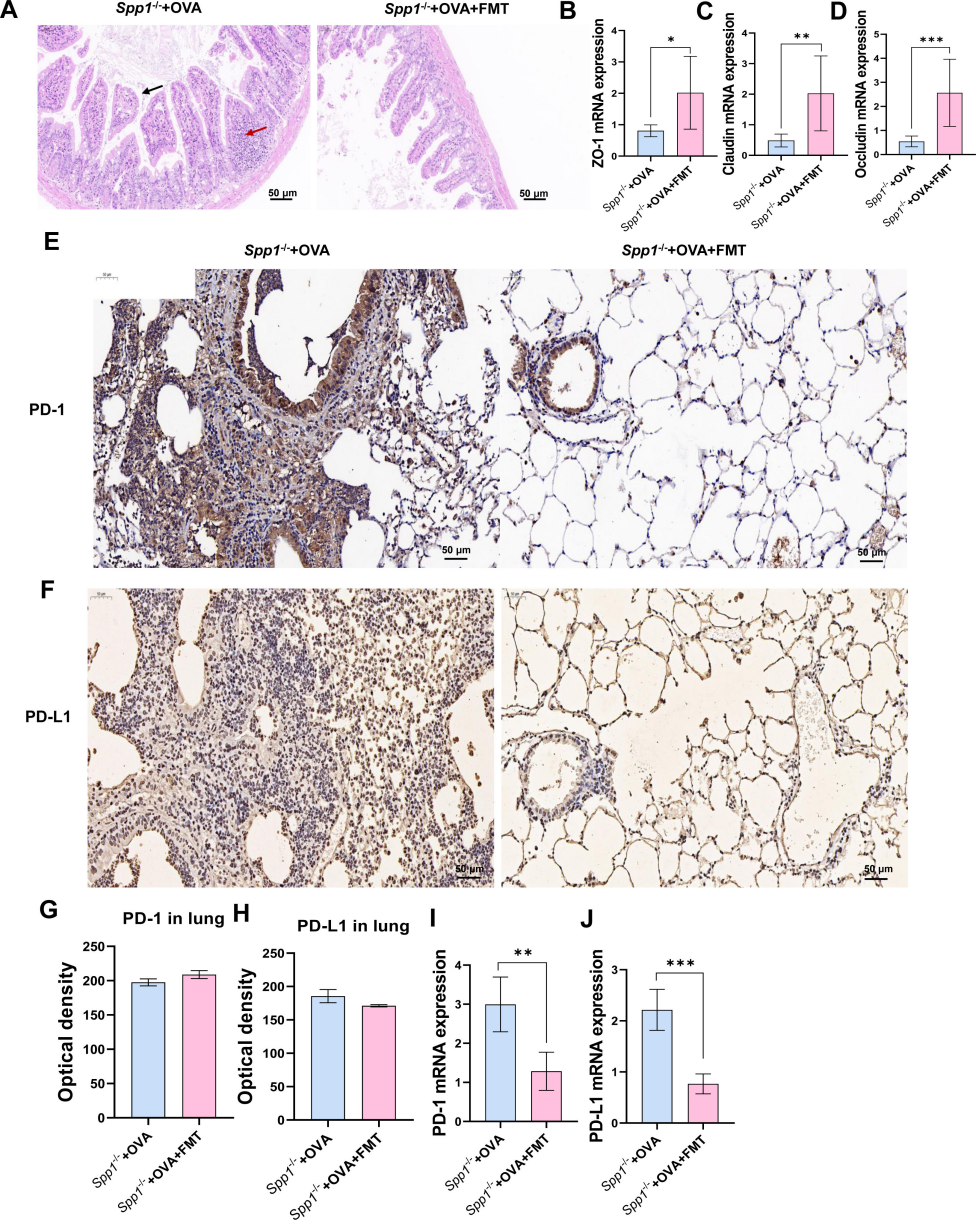
FMT reduces gut permeability and affects the expression of PD-1 and PD-L1 in OPN knockout asthma mice. (**A**) Hematoxylin and eosin staining of small intestine (200×). (**B–D**) ZO-1, claudin, and occludin mRNA expression in the small intestine by qPCR. (**E–H**) Immunohistochemistry examination (200×) and optical density of PD-1 and PD-L1 in the lung. (**I and J**) PD-1 and PD-L1 mRNA expression in the lung by qPCR. The data are the means ± SD; *P < 0.05, **P < 0.01, ***P < 0.001. Figure 2A and K (Spp1^−/−^+OVA group) and panels A, E, and F (Spp1^−/−^+OVA group) use the same images to present different analytical perspectives.

To assess the impact of the microbiota on intestinal barrier function in Spp1^−/−^ mice, we stained the small intestine for H&E (Fig. 6A) and examined the expression of tight junction-associated proteins and found disruption of small intestinal villi (as indicated by the black arrows) and infiltration of inflammatory cells (as indicated by the red arrows) in the Spp1^−/−^+OVA group and attenuated lesions in the small intestine of the Spp1^−/−^+OVA+FMT group. Moreover, the expression of ZO-1 (Fig. 6B), claudin (Fig. 6C), and occludin (Fig. 6D) was significantly higher in the Spp1^−/−^+OVA+FMT group compared with the Spp1^−/−^+OVA group. The results suggest that microorganisms improve intestinal barrier function in OPN knockout mice.

### FMT increases the PD-1/PD-L1 expression in OPN knockout asthma mice

To clarify the effects of the microbiota on Treg cell function in Spp1^−/−^ mice and their mechanisms, we examined PD-1 and PD-L1 expression by immunohistochemistry and qPCR. Compared with the Spp1^−/−^+OVA group, immunohistochemical results showed no significant differences in the amounts of PD-1 and PD-L1 in lung tissue (Fig. 6E through H), but their mRNA expression was significantly reduced (Fig. 6I and J).

### FMT treatment alleviates gut and lung microbiota dysbiosis of the OPN knockout asthma model

**Fig 7:**
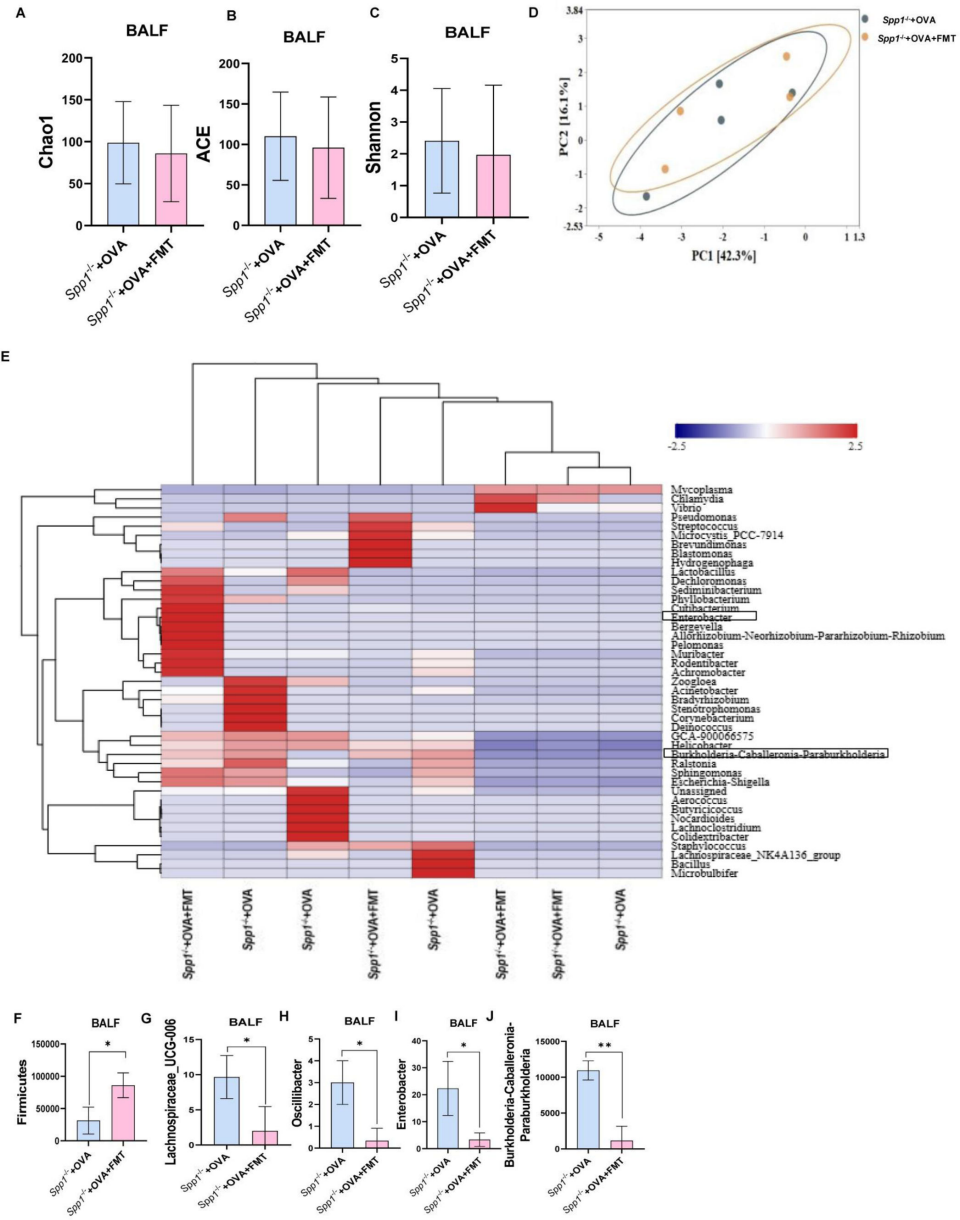
FMT alleviates microbiota dysbiosis of BALF in OPN knockout asthma mice. (**A–C**) Chao1, ACE, Shannon index, and average. (**D**) PCA. (**E**) Composition of microbiota at genus level. (**F**) Phylum-bacteria. (**G–J**) Bacterial in genus levels. *P < 0.05. L, lung; In, intestine; Inf, INF-γ.

Recently, accumulating reports have found microbiota play a key role in the pathogenesis of asthma, microbiota can modify the host's response to allergens through direct interactions with the host or by influencing the host immune system via metabolites (41). Our study identified concurrent lung and gut microbiota dysbiosis in OPN-deficient mice with allergic asthma, suggesting that OPN deficiency may exacerbate asthma by disrupting microbial-immune crosstalk. To explore how FMT administration protects the OPN knockout asthma mice model through modulating microbiome community structures, we carried out 16S RNA sequencing on fecal and BALF samples of mice from different groups. There was no significant difference in Chao1, ACE, and Shannon index between the two groups (Fig. 7A through C). Moreover, the structures of the Spp1^−/−^+OVA+FMT group separated from the Spp1^−/−^+OVA group, and the composition of the microbiota was also different for the two groups (Fig. 7D and E). To further identify the critical bacteria, we compared the relative abundances of microbes at phylum and genus levels among different groups. At the phylum level, *Firmicutes* showed a significant increase in the Spp1^−/−^+OVA+FMT group compared to the Spp1^−/−^+OVA group (Fig. 7F). Compared to the Spp1^−/−^+OVA group, the genera *Lachnospiraceae_UCG-006*, *Oscillibacter*, *Enterobacter*, and *Burkholderia-Caballeronia-Paraburkholderia* showed significantly decreased relative abundances in the Spp1^−/−^+OVA+FMT group (Fig. 7G through J).

**Fig 8:**
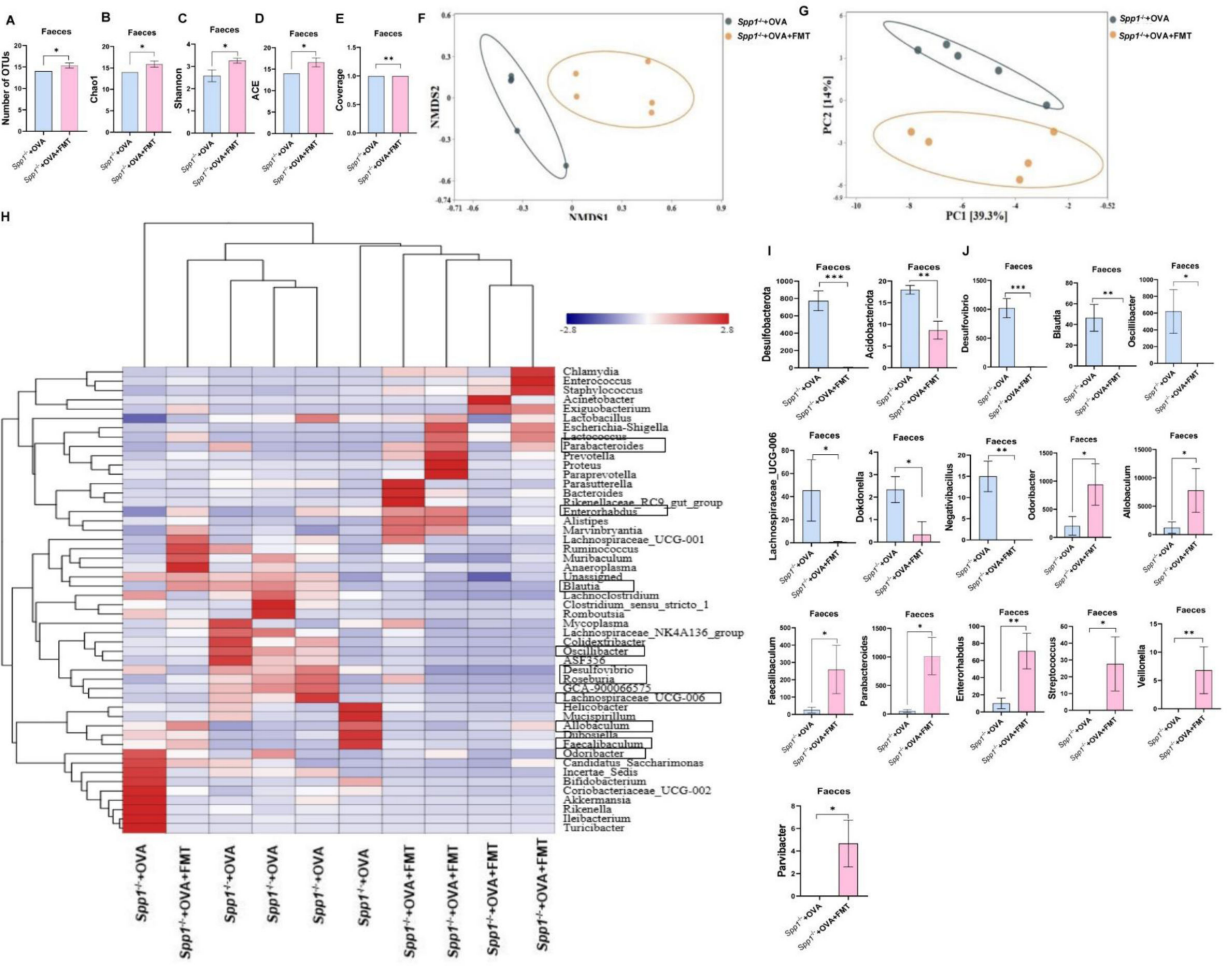
FMT alleviates microbiota dysbiosis of feces in OPN knockout asthma mice. (**A**) Number of OTUs. (**B–E**) Chao1, ACE, Shannon index, and average. (**F and G**) PCA and NMDS. (**H**) Composition of microbiota at genus level. (**I, J**) Bacterial in phylum and genus levels. *P < 0.05. L, lung; In, intestine; Inf, INF-γ.

To assess the diversity and richness of bacterial species of the fecal microbiome, the alpha-diversity analysis was performed. Increased number of OTUs (Fig. 8A), Chao1 (Fig. 8B), Shannon index (Fig. 8C), ACE (Fig. 8D), and coverage (Fig. 8E) were present in the Spp1^−/−^+OVA+FMT group compared to the Spp1^−/−^+OVA group. In addition, beta diversity measurements showed similarity in the composition of the two groups of microbiomes. Non-metric multidimensional scaling (NMDS) and PCA showed that the structures of the Spp1^−/−^+OVA+FMT group separated from the Spp1^−/−^+OVA group (Fig. 8F and G). Analysis of the composition of the microbial community revealed that the microbiome composition and relative abundance of the Spp1^−/−^+OVA+FMT group was different from those of the Spp1^−/−^+OVA group at the genus level (Fig. 8H). We analyzed the relative abundances of microorganisms at different taxonomic levels between the two groups in order to further identify the critical bacteria that contributed to the development of asthma in OPN knockout mice. At the phylum level, *Desulfobacterota* and *Acidobacteriota* had lower abundance in the Spp1^−/−^+OVA+FMT group than the Spp1^−/−^+OVA group (Fig. 8I). At the genus level, compared with the Spp1^−/−^+OVA group, the relative abundance of *Desulfovibrio*, *Blautia*, *Oscillibacter*, *Lachnospiraceae_UCG-006*, *Dokdonella*, and *Negativibacillus* was significantly reduced, and the relative abundance of *Odoribacter*, *Allobaculum*, *Faecalibaculum*, *Parabacteroides*, *Enterorhabdus*, *Streptococcus*, *Veillonella*, and *Parvibacter* was significantly increased in the Spp1^−/−^+OVA+FMT group (Fig. 8J). These data indicate that FMT improves gut and lung microbiome disorder in the OPN knockout asthma model.

## DISCUSSION

The role of OPN in asthma has gained increasing attention in recent years, with a strong correlation between OPN and asthma (42). OVA-induced allergic inflammation was increased in OPN knockout asthmatic mice, and allergic inflammation-induced tissue damage and microvascular leakage may be the result of increased influx of immune cells. Cationic granule proteins released by eosinophils and neutrophils, extracellular histones released by damaged cells, proteases, and inflammatory mediators increase microvascular leakage and cause structural damage (43). Basophil-derived IL-4 promotes lung inflammation by activating ILC2s in the lung to recruit eosinophils (44). An altered balance between Th1 and Th2 cytokines is responsible for a variety of immunoinflammatory disorders such as asthma (45). A significant decrease in the expression of the Th1 polarization factor IFN-γ and a significant increase in the expression of the Th2 polarization factors IL-4 and GATA-3 in Spp1^−/−^+OVA mice, which suggests a lack of OPN, increases the Th2-polarized environment in asthma. In the Th2-polarized environment characteristic of allergic asthma, high levels of IL-4 produced by locally infiltrating innate lymphoid cells and helper T cells promote the acquisition of an alternatively activated M2a phenotype in macrophages, with myriad effects on the local immune response and airway structure (46). The tissue damage was significantly more severe in Spp1^−/−^+OVA mice than in OVA mice, suggesting important roles for OPN in tissue protection. The protective effect of OPN has been shown in other disease models. For example, in models of reperfusion injury in the heart, liver, and kidney, OPN is essential for reducing tissue damage (47).

Compelling evidence indicated that Treg cell function was involved in the pathogenesis of asthma (48). It has been found that reducing airway inflammation by increasing Treg response is able to exert an anti-asthmatic effect (49). As the primary transcription factor of Treg cells, FOXP3 plays a pivotal role in regulating both the function and plasticity of these cells. The observed downregulation of FOXP3 expression in the lungs of Spp1^−/−^+OVA mice likely contributed to their exacerbated airway inflammation phenotype. Alteration of the PD-1/PD-L1 pathway can modulate Treg/Th17 balance in asthma (50). To elucidate the mechanism by which OPN affects asthma, we examined the expression of PD-1/PD-L1 in the small intestine and lungs of OPN knockout mice and found significant increases in both PD-1 and PD-L1. More importantly, the expression of PD-1 and PD-L1 was significantly negatively correlated with the FOXP3 expression. Like the study, treatment with anti-PD-1 posed protective effects on asthma models (50). This result suggests that OPN deletion aggravates asthma by reducing the expression of FOXP3 by elevating PD-1/PD-L1 expression.

Emerging evidence indicates that asthma risk and disease severity are closely linked to dysregulated immune responses and intestinal microbial dysbiosis, which collectively contribute to impaired mucosal barrier function (51). As a result, we examined the changes in intestinal barrier function and found that Spp1^−/−^+OVA mice had disruptions in small intestinal structure, breakage of intestinal villi, and massive infiltration of inflammatory cells. In addition, the expression levels of intestinal barrier function-related proteins were significantly reduced. The impairment of intestinal barrier function facilitates microbial translocation, potentially triggering both systemic and local inflammatory responses that disrupt immune homeostasis (52).

There is an intimate association of microbial communities with host immune development and the development of allergic airway inflammation. Data from multiple human clinical studies demonstrate an association between alterations in the lung microbiota and various asthma phenotypes (53). In addition, it is now recognized that the gut microbiota plays an important role in asthma. Perturbation of the gut microbiota is known to have an indirect physiologic consequence on remote anatomic sites including the lung. We examined the microbiota of the gut and lungs of wild-type and OPN knockout mice and found that the microbes in the gut and lungs of Spp1^−/−^+OVA mice were different in composition from those of OVA mice. The abundance and diversity of microbes in the gut and lungs of Spp1^−/−^+OVA mice were reduced. Beneficial bacteria such as *Bacteroidetes* were decreased, and *Epsilonbacteraeota* increased. Research found that the relative abundances of phylum Bacteroidetes were associated with decreased eosinophilic inflammation (54). *Epsilonbacteraeota* are tolerant of bile acids and promote hepatic inflammation. The increased abundance of *Epsilonbacteraeota* observed in the BALF of Spp1^−/−^+OVA mice may help to increase the production of inflammatory cytokines in response to induced airway inflammation (55). At the genus level, our analysis revealed a significant reduction in Lactobacillus and its phylogenetically related OTU6 in Spp1^−/−^+OVA mice. Probiotic bacteria, such as *Lactobacillus* species, have shown anti-allergic effects in various mouse and human studies. Both human and murine studies have demonstrated that oral administration of *Lactobacillus* strains modulates immune responses by enhancing Th1 cytokine production while suppressing Th2 cytokine expression. This immunomodulatory effect is associated with reduced allergy-related immunoglobulins and inflammatory cell infiltration. Clinically, *Lactobacillus* supplementation has shown therapeutic benefits, including decreased activity limitations, reduced rhinitis episodes, and prolonged symptom-free periods in asthma and allergic rhinitis patients (56). *Lactobacillus* was significantly negatively associated with total cell count in BALF, IL-17F in the lung, IL-4 in the lung, and PD-L1 expression in the small intestine; it was positively associated with claudin in the small intestine. This suggests that decreased *Lactobacillus* abundance may account for increased inflammation, reduction of FOXP3 expression, and damage to intestinal barrier function in OPN knockout asthmatic mice. Similarly*,* Spp1^−/−^+OVA mice showed reduced abundance of short-chain fatty acid (SCFA)-producing commensal bacteria, including *Roseburia*, *Lachnoclostridium*, and *Parabacteroides*. These bacterial taxa may serve as potential biomarkers for disease progression or therapeutic targets for microbiota restoration strategies (57). Notably, our study reveals that Spp1^−/−^+OVA mice exhibit a distinct gut microbiota profile characterized by decreased abundance of *Lachnospiraceae-UCG-001* alongside increased populations of *Helicobacter*, *Burkholderia-Caballeronia-Paraburkholderia*, and *Dechloromonas*. Taken together, we can conclude that OPN knockout in asthma increases airway inflammation and impacts Treg cell function primarily by reducing beneficial and increasing harmful bacteria in BALF.

We have also observed significant changes in bacteria in the gut microbiota. We found that *Acidobacteriota* increased in Spp1^−/−^+OVA mice. No previous studies have been reported. As with the BALF, fecal *Lactobacillus* and *Bifidobacterium* in Spp1^−/−^+OVA mice were also lower. However, *Bifidobacterium* shows several beneficial properties to the hosts. *Bifidobacterium breve* B632 significantly reduced the frequency of asthma exacerbations (58). Moreover, *Bifidobacterium infantis* has been shown to attenuate allergy airway inflammation by modulating immune responses through promoting Th1 while simultaneously suppressing Th2-mediated pathways. Research found that in OVA-induced Spp1^−/−^ mice, the relative abundance of *Allobaculum* is reduced. As a short-chain fatty acid (SCFA)-producing bacterium, the decreased abundance of *Allobaculum* promotes inflammatory responses and suppresses FOXP3 expression (59), thereby exacerbating intestinal inflammation. In support of this interpretation, *Allobaculum* showed a significant positive correlation with FOXP3 and IFN-γ in the lungs and a significant negative correlation with IL-4 levels in the BALF, GATA-3 expression in the lungs, and PD-1 expression in the small intestine. We found a significant decrease in *Acinetobacter* abundance in Spp1^−/−^+OVA mice. A recent study found that it had asthma protection operating through IL-6-mediated epigenetic activation of IL-10 production and with associated effects on the intestinal microbiome (60). Our study detected reduced *Paraprevotella*, *Muribaculum,* and increased *Desulfovibrio*, *Enterococcus*, *Negativibacillus*, *Dokdonella*, *Christensenellaceae_R-7_group*, *Escherichia-Shigella*, and *Lachnospiraceae FCS020* in the Spp1^−/−^+OVA mice. To our knowledge, this is the first time that the changes in the abundance of these bacteria have been revealed through 16S RNA sequencing in the OPN knockout asthma model, which may serve as new biomarkers for asthma. In addition to BALF, we found a significant reduction in the abundance of *Parabacteroides* in the intestine. We found that the relative abundance of *Blautia* significantly increased in Spp1^−/−^+OVA mice. Fu et al. reported that *Blauti*a was positively correlated with asthma (61); furthermore, studies have found that there is a lack of binding between IgA and *Blautia* in children with asthma. This lack of binding is associated with poor asthma control and an increased risk of severe asthma (62).

To test our hypothesis that gut microbiota dysbiosis is necessary in OPN knockout asthma genesis, we further monitored the effects of FMT in mice of the OPN knockout asthma model, showing that the OPN knockout asthma mice receiving gut microbiota exhibited both reduced airway inflammation and tissue damage. In addition, intestinal barrier function was improved. Moreover, microbiota disorders were found to improve in the gut and the lung after FMT treatment, suggesting the importance of the gut-lung axis. Furthermore, after FMT treatment, the microbiota disorders in the intestine and lungs were improved, suggesting that the intestinal microbiota plays a role in the pulmonary microbiota dysregulation of asthma caused by OPN deficiency through the gut-lung axis. However, the protective effect of FMT on OPN-deficient asthmatic mice and its possible mechanisms have been rarely investigated. Based on the important role of microbiota dysbiosis in the development of asthma, the present study provides insights into the possible mechanisms in addition to evaluating the protective effects of FMT treatment in OPN-deficient asthmatic mice. In our study, microbial disorders in the small intestine and lungs of OPN-deficient asthmatic mice were improved after FMT treatment, PD-1/PD-L1 levels were significantly reduced, and FOXP3 expression was significantly decreased. Correlation analysis revealed that the expression levels of PD-1/PD-L1 were significantly negatively correlated with the expression of FOXP3. Therefore, FMT regulates the PD-1/PD-L1 axis through the microbiota to impact Treg cell function to alleviate asthma in OPN-deficient mice.

Taken together, our study suggests that OPN deficiency exacerbates asthma via the gut-lung axis. OPN deficiency caused airway inflammation, increased tissue damage, disturbed gut and lung microbiota, and impaired intestinal barrier function in asthmatic mice. In addition, PD-1 and PD-L1 expression was found to be increased, and FOXP3 expression decreased in the small intestine and lungs, which further exacerbated airway inflammation in asthma. Thus, we demonstrated that OPN may play a protective role in asthma by a mechanism that may alleviate asthma by modulating the microbiota to influence the PD-1/PD-L1 pathway to increase Treg cell function. Moreover, our study reveals the protective effect of FMT on an OVA-induced OPN knockout asthma mouse model. Mechanistic studies showed that FMT reversed gut and lung microbial dysbiosis and protected the OPN knockout asthmatic mice, in which increasing FOXP3 expression by improving the gut barrier function and decreasing PD-1/PD-L1 signaling pathway in the gut and lungs may play an important role. Furthermore, it demonstrates that the potential effects of OPN on asthma are closely related to the microbiota. However, the long-term impacts are not yet clear, and further research is needed.

The present study also has some limitations: further studies are needed to clarify whether the causal relationship is that the disturbed gut microbiota and impaired intestinal barrier function further affect lung function and lung microbiota or whether the airway inflammation due to asthma affects the lung microbiota. Furthermore, it is necessary to further study how the microbiota in the OPN knockout asthma model affects intestinal barrier function through the PD-1/PD-L1 pathway. Moreover, in this study, we did not backcross WT animals with Spp1 knockout (KO) animals to verify the role of the *Spp1* gene in the regulation of bacterial colonization. In future studies, we will further validate the results by backcrossing. 16S rRNA sequencing lacks insights into functional aspects. In future studies, we plan to employ shotgun metagenomic sequencing and metatranscriptomic analysis to more comprehensively reveal the functional significance of microbial changes and explore their relationship with the pathogenesis of asthma.

In conclusion, this study confirmed the protective role and underlying mechanism of OPN in asthma. Also, we used FMT to demonstrate the important role of gut and lung microbiota in OPN knockout mice in an ovalbumin-induced asthma model. The study provides clinicians with new insights into asthma mechanisms and can also lead to new ideas for asthma treatment.

## Data Availability

The data sets generated during and/or analyzed during the study are available from the corresponding authors on reasonable request.

## SUPPLEMENTAL MATERIAL

Fig. S1 msystems.00389-25-s0001.docx Effects of OPN knockout on airway remodeling and airway hyperresponsiveness in asthmatic mice.

Fig. S2 msystems.00389-25-s0002.docx Composition of microbiota at genus level in BALF and correlation between *Lactobacillus* and clinical indicators.

Fig. S3 msystems.00389-25-s0003.docx Composition of microbiota at genus level in feces and correlation between *Allobaculum* and clinical indicators.

ASM does not own the copyrights to Supplemental Material that may be linked to, or accessed through, an article. The authors have granted ASM a non-exclusive, world-wide license to publish the Supplemental Material files. Please contact the corresponding author directly for reuse.
