# Supplementary material for: Osteopontin protects from ovalbumin-induced asthma by preserving the microbiome and the intestinal barrier function
Source: mSystems. 2025 May 22;10(6):e00389-25. doi: 10.1128/msystems.00389-25 (PMC12172459; doi:10.1128/msystems.00389-25)
Supplement: Fig. S1 — Effects of OPN knockout on airway remodeling and airway hyperresponsiveness in asthmatic mice. [file msystems.00389-25-s0001.docx]

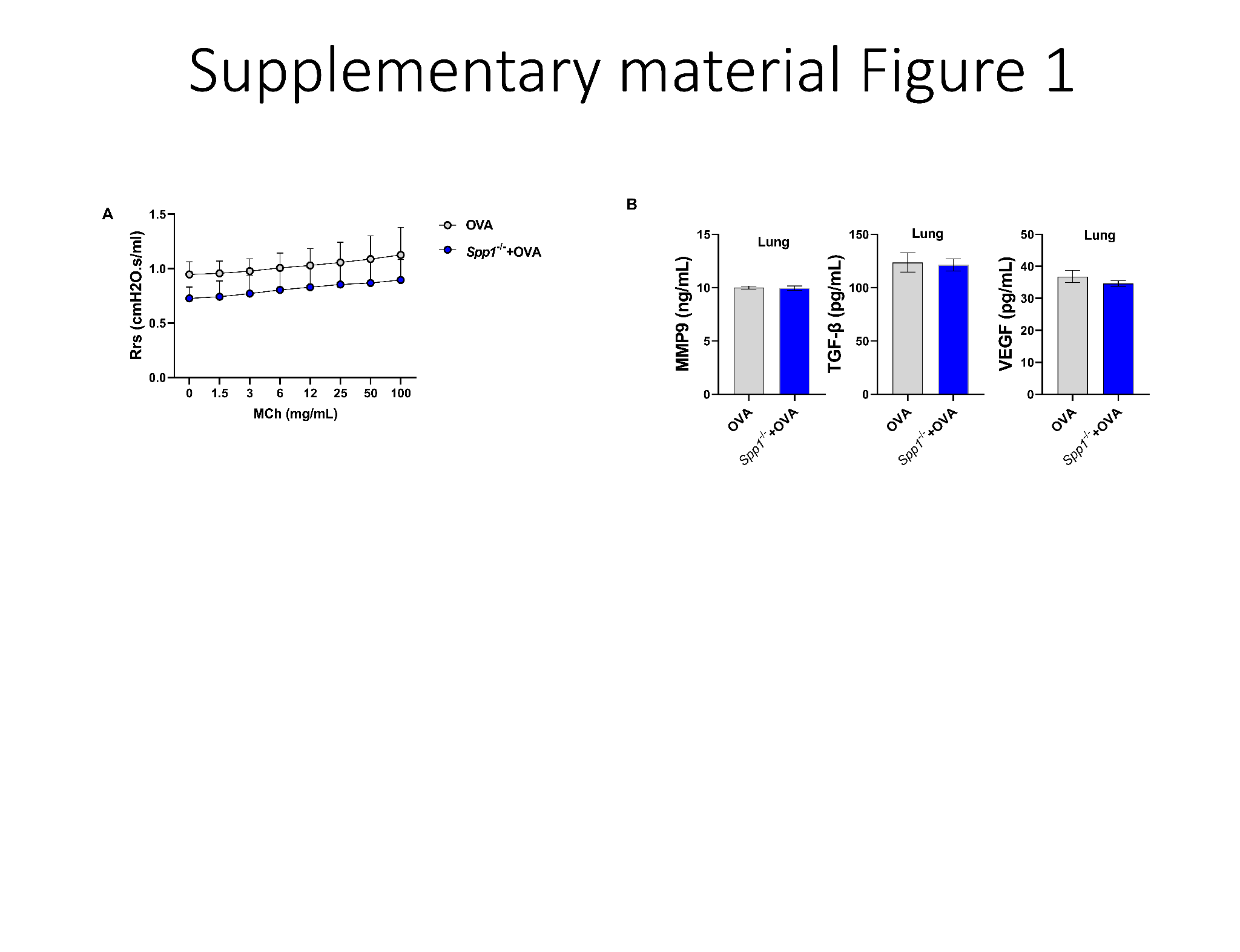


Figure_S1. Effects of OPN knockout on airway remodeling and airway hyperresponsiveness in asthmatic mice. (A) Airway hyperresponsiveness. (B) Markers of airway remodeling. The data are the means ± SD; ^*^*P* < 0.05, ^**^*P* < 0.01, ^***^*P* < 0.001. OPN, osteopontin; MMP9, matrix metallopeptidase-9; TGF-β, transforming growth factor-β; VEGF, vascular endothelial growth factor.
