# Supplementary material for: Osteopontin protects from ovalbumin-induced asthma by preserving the microbiome and the intestinal barrier function
Source: mSystems. 2025 May 22;10(6):e00389-25. doi: 10.1128/msystems.00389-25 (PMC12172459; doi:10.1128/msystems.00389-25)
Supplement: Fig. S2 — Composition of microbiota at genus level in BALF and correlation between Lactobacillus and clinical indicators. [file msystems.00389-25-s0002.docx]

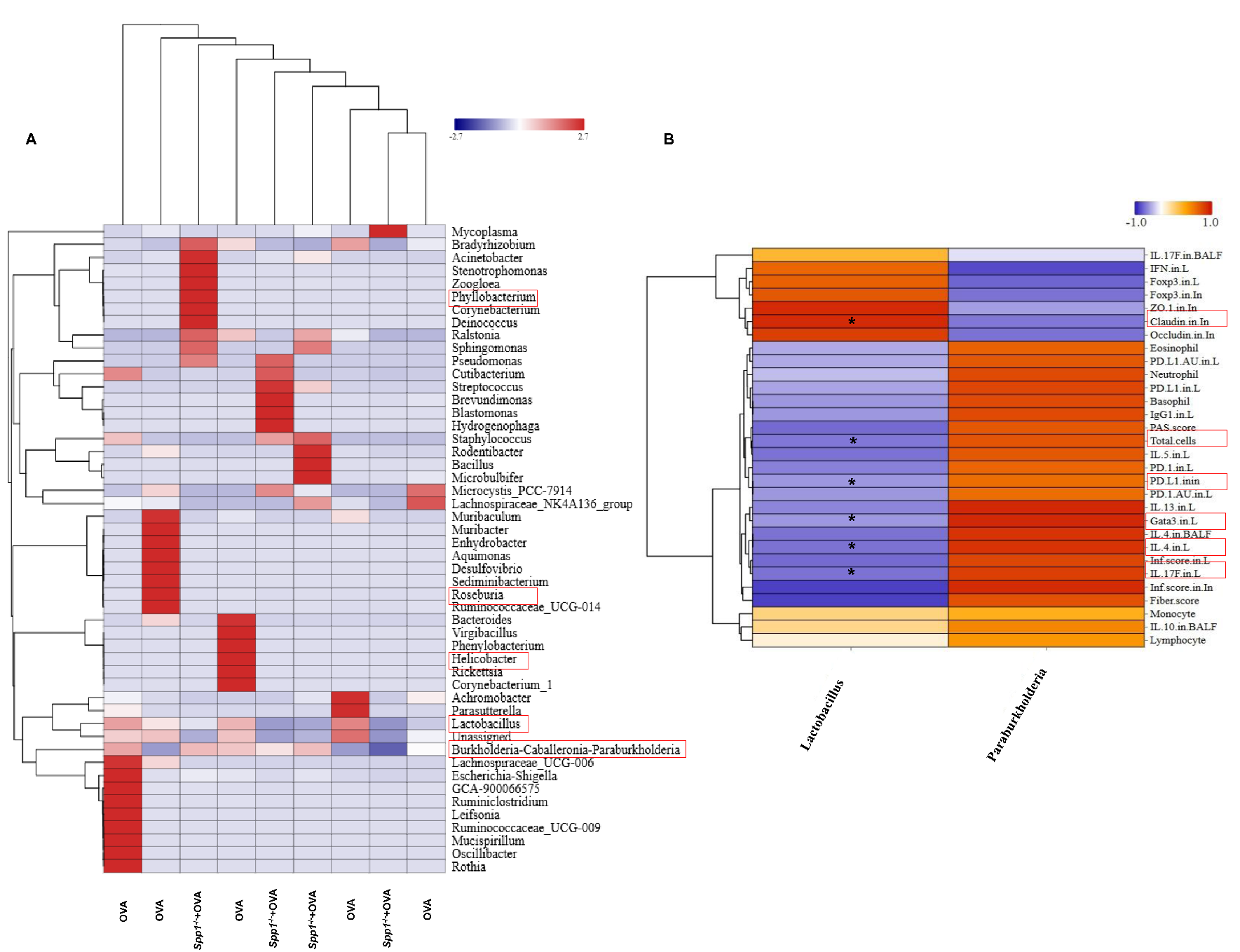


Figure_S2. (A) Composition of microbiota at genus level in BALF. (B) Correlation between *Lactobacillus* and clinical indicators.
