# Supplementary material for: Osteopontin protects from ovalbumin-induced asthma by preserving the microbiome and the intestinal barrier function
Source: mSystems. 2025 May 22;10(6):e00389-25. doi: 10.1128/msystems.00389-25 (PMC12172459; doi:10.1128/msystems.00389-25)
Supplement: Fig. S3 — Composition of microbiota at genus level in feces and correlation between Allobaculum and clinical indicators. [file msystems.00389-25-s0003.docx]

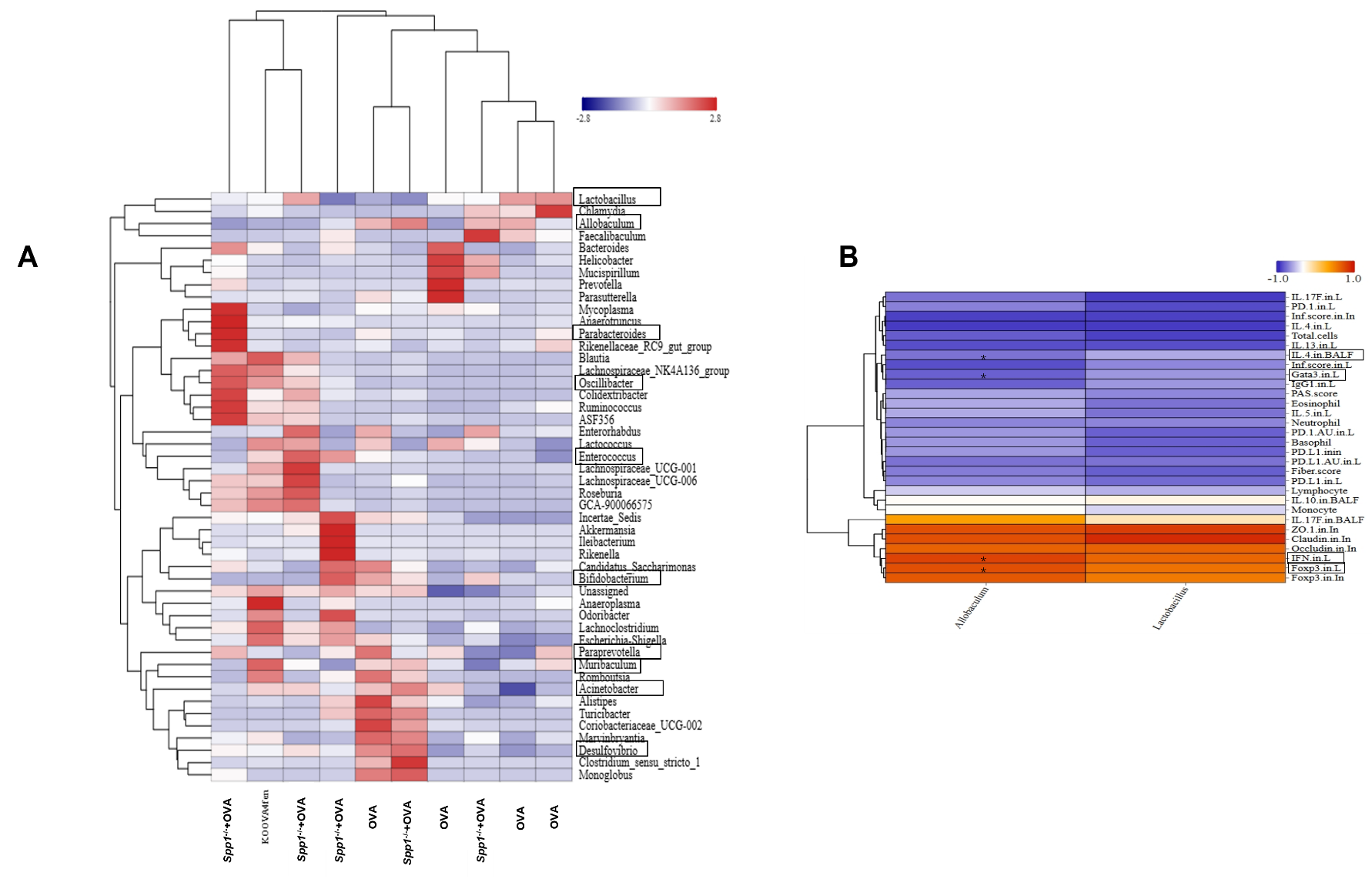


Figure S_3. (A) Composition of microbiota at genus level in feces. (B) Correlation between *Allobaculum* and clinical indicators. ^*^*P* < 0.05.
